# The costs of offering HPV-testing on self-taken samples to non-attendees of cervical screening in Finland

**DOI:** 10.1186/s12905-015-0261-7

**Published:** 2015-11-05

**Authors:** Anni Virtanen, Ahti Anttila, Pekka Nieminen

**Affiliations:** Mass Screening Registry, Finnish Cancer Registry, Unioninkatu 22, FI-00130 Helsinki, Finland; Department of Obstetrics and Gynecology, Kätilöopisto Hospital, Helsinki University Central Hospital, Helsinki, Finland

**Keywords:** Cervical cancer, Screening, Self-sampling, HPV-testing, Cost-evaluation

## Abstract

**Background:**

Offering self-sampling to non-attendees of cervical screening increases screening attendance.

**Methods:**

We used observations from two Finnish studies on the use of self-sampling among the non-attendees to estimate in a hypothetical screening population of 100,000 women the possible costs per extra screened woman and costs per extra detected and treated CIN2+ with three intervention strategies; 1) a primary invitation and a reminder letter, 2) a primary invitation and a mailed self-sampling kit and 3) two invitation letters and a self-sampling kit. The program costs were derived from actual performance and costs in the original studies and a national estimate on management costs of HPV related diseases.

**Results:**

The price per extra participant and price per detected and treated CIN2+ lesion was lower with a reminder letter than by self-sampling as a first reminder. When self-sampling was used as a second reminder with a low sampler price and a triage Pap-smear as a follow-up test for HPV-positive women instead of direct colposcopy referral, the eradication of a CIN2+ lesion by self-sampling was not more expensive than in routine screening, and the addition of two reminders to the invitation protocol did not increase the price of an treated CIN2+ lesion in the entire screened population.

**Conclusions:**

As a first reminder, a reminder letter is most likely a better choice. As second reminder, the higher costs of self-sampling might be compensated by the higher prevalence of CIN2+ in the originally non-attending population.

## Background

A high coverage and participation rate throughout the program is of primary importance to the success of a screening program. The participation rate of the organized cervical cancer screening program in Finland is currently at 70 % with a decreasing trend [1]. Non-attenders contribute significantly to cervical cancer incidence and mortality [2, 3].

Ways to improve the attendance rate include personal invitations to screening, preferably with a pre-assigned date and place for the screening appointment [4–6], reminder letters or telephone reminders to non-attendees [7–15], and, when appropriate, a GPs signature on the invitation [6, 16].

As a recent approach, offering self-sampling for HPV-testing for the non-attendees of cervical screening might increase screening attendance [13, 17–22]. In Finland, offering self-sampling to non-attendees after primary invitation (i.e. as a first reminder) increased total attendance by 17 % and the yield of detected moderate or more severe cervical intraepithelial lesions (CIN2+) by 13 % [12]. When self-sampling was offered as a second reminder after two invitation letters (primary invitation and a reminder letter), it increased total attendance by 4 %, CIN2+ yield by 8 %, and yield of severe cervical lesions or cancer (CIN3+) by 9 % [14]. However, the costs to obtain these effects must also be considered before making recommendations for routine practice.

Here we estimated differences in the program costs of using self-sampling vs. using a reminder letter as a first reminder for non-attendees after primary invitations, and further, what could be the cost of using self-sampling as a second reminder after two invitation letters.

## Methods

In the Finnish cervical screening program, all women aged 30–60 are invited to give a screening sample by personal invitations in 5-year intervals. In addition, those women belonging to a risk group based on the previous abnormal screening results or anamnestic data are invited within 1–2 years after the previous screening round [23]. The responsibility to organize screening lies within individual municipalities. Participation rate in the organized program is currently less than70 % [1]. Extensive opportunistic screening occurs beside the organized program [24].

This cost analysis evaluation is based on two Finnish studies on the use of self-sampling among non-attendees of routine cervical screening, described in detail elsewhere [12–14].

The first study was conducted in 2008–2009 as a part of the routine screening program of the city of Espoo. It evaluated the effects of using a self-sampling test as a first reminder for non-attendees in a randomized setting in comparison to a reminder letter. In 2009, approximately half of the reminder letter arm non-attendees further received a self-sampling test as a second reminder [12].

The second study assessed the effect of using self-sampling as a second reminder (i.e. among non-attendees after a primary invitation and a reminder letter) in a non-randomized setting in 31 Finnish municipalities in 2011–2012 [14].

**Fig. 1:**
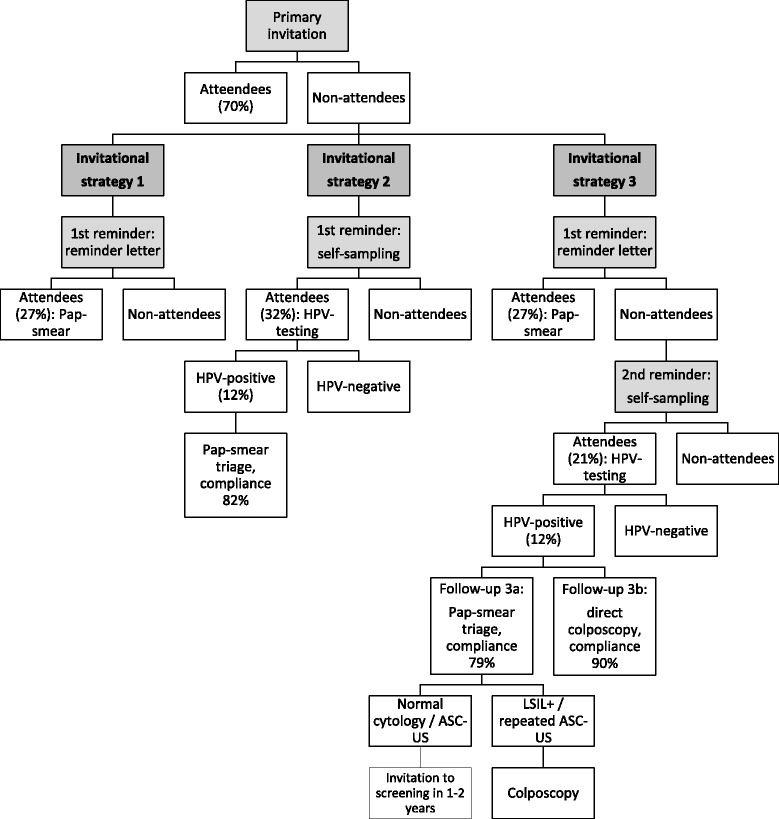
Evaluated invitational and follow-up strategies in a population of 100,000 women to be screened

Here we use the average participation rates, referral rates and precursor lesion yields observed in the studies, to estimate in a hypothetical population of 100,000 women invited to screening the costs per extra screened woman and costs per extra detected and treated CIN2+ by three different invitation strategies: 1) a primary invitation and a reminder letter, 2) a primary invitation and a mailed self-sampling kit and 3) a primary invitation, a reminder letter and a mailed self-sampling kit as a second reminder (with two different follow-up strategies for HPV-positive women). A flow chart of invitational strategies is shown in Fig. 1.

The cost per extra screened woman was calculated by including only the costs of the invitational system, primary screening test and, in the case of self-sampling, possible triage testing. The cost per one detected and treated CIN2+ case included all costs from the invitational system and primary testing to colposcopic investigations, treatment and subsequent follow-up testing. As no outcome information was available from the original studies, CIN2+ was used as a proxy for the potential of increasing the impact of the program. The cost per one extra eradicated CIN2+ case by each of the reminders was compared to the cost of eradicating a CIN2+ case in routine screening under the speculation that if the price of an eradicated CIN2+ lesion in routine screening is regarded acceptable and the price per lesion does not increase after adding one to two reminders to the protocol, the cost increase might be acceptable.

The estimate was calculated assuming 70 % attendance rate by primary invitation, use of Pap-smear as a primary screening test and current national detection rates of CIN lesions among screening participants as baselines [1].

Participation rate with self-sampling as first reminder was set at 32 % and as second reminder at 21 % [12, 14]. Participation with reminder letter was estimated at 27 % as observed for reminder letters sent with pre-assigned appointments [12, 14], but additional analysis was performed to estimate the effect of open invitations with lower participation rate of 14 % [14]. HPV-positivity rate was set at 12 % as observed for Hybrid Capture 2 (HC2; Qiagen, Gaithersburg, MD, USA) in the studies.

### Costs of screening

Only direct costs were considered in this analysis. The cost estimates for Pap-smears, diagnostic colposcopies and management of CIN2+ diseases were derived from a previous evaluation on the costs of prevention and management of HPV-related diseases in Finland that used mean national prices [25]. The costs were evaluated from the health care provider perspective and were here presented in 2012 prices.

The cost of a Pap-smear includes sample taking, analysis and registration. The cost of HPV-analysis was estimated at 20 euros, but to accommodate for the differences in cost with other analysis methods and decreasing market prices, additional analysis was conducted with cost estimates of 15 and 30 euros. The cost of a CIN case includes diagnostic and treatment procedures and follow-up after treatment, and cost of a CIN2+ case is here presented as the average of the cost of a CIN2 and a CIN3 case. The cost of a case referred to colposcopy that resulted in normal results or minor cytological changes without CIN diagnosis includes diagnostic procedures and follow-up.

The cost of a primary screening invitation includes the costs of identifying the screening population from the Population Register Centre and cost of the letter itself. The cost of a reminder letter includes only the cost of the letter itself. A common practice is to use an online program for appointment scheduling and mailing the invitations and identifying the non-attending population in need of re-invitations.

Cost estimates for self-sampling were estimated using a price of 2.0 euros for the sampling device and the actual mean costs in the study of 2011–2012 for other related costs (other materials, logistics costs and mailing). A higher price of 6.5 euros for the sampling device was used in additional analysis.

Both original studies included an opt-out option for the self-sampling offer. The opt-out rate was 15 % in both studies. Calculations assuming 15 % opt-out rate were made as an additional analysis.

### Follow-up after an HPV-positive self-taken sample

In the evaluation of self-sampling as a first reminder, the estimate was based on a Pap-smear triage follow-up with 82 % follow-up compliance rate [12]. In the original randomized study of self-sampling vs. reminder letter as a first reminder the CIN yields after respective interventions differed somewhat between arms, but the numbers were too small for reliable comparisons in the background cancer risk of the participants by respective interventions or differences in clinical sensitivity by respective testing methods. Thus, in this analysis the CIN detection rate is assumed to be similar in both interventions. However, as there has been suggestions that self-sampling might attract women with higher risk for cancer [22, 26], additional analysis was conducted assuming 20 % and 50 % higher detection rates of CIN2+ by self-sampling than by reminder letter.

In the evaluations of using self-sampling as a second reminder, estimates were calculated for two different follow-up strategies for women with a hrHPV-positive result in self-taken sample; for the strategy of inviting all hrHPV-positive women for a triage Pap-smear, and for the strategy of referring all of them directly for colposcopy and biopsies (Fig. 1). Compliance to follow-up in Pap-smear triage was estimated to be 79 % [12], but a lower rate of 70 % [14] was used in additional analysis, and compliance to direct colposcopy follow-up was set at 90 % [12, 14].

For the break-down of resource required for screening a population of 100,000 women in the different invitation strategies and a more precise rationale on the unit numbers used in the estimate, see Table 1.

**Table 1:** Resource required for screening a population of 100,000 women in three different invitation strategies (costs included per strategy marked by ‘x’). Estimates on self-sampling as a second reminder with two different follow-up strategies after a hrHPV-positive self-taken sample. For total costs per invitational strategy, see Table 2. Flow-chart on invitational strategies as Fig. 1

Invitational strategy					Unit cost (€)	Units needed	Total cost (€)		
1	2	3a	3b					Rationale for units needed	Reference
x	x	x	x	Primary invitation	1.00	100,000	100,000		
x	x	x	x	Pap-smear for attendees (70 %)	29.80	70,000	2,086,000		http://www.cancerregistry.fi/statistics, [25]
				Diagnostic colposcopy		630		Average national rate of referrals for diagnostic confirmations, 0.9 % of attendees	http://www.cancerregistry.fi/statistics
x	x	x	x	No CIN diagnosis^a^	1,047.10	350	366,485		[25]
x	x	x	x	CIN1 case, inlc. treatment and follow-up^b^	1,863.40	70	130,438	Average national yield of CIN1/CIN2+ lesions, 0.1 %/0.3 % of attendees	http://www.cancerregistry.fi/statistics, [25]
x	x	x	x	CIN2+ case, inlc. treatment and follow-up^b^	2,512.00	210	527,520		http://www.cancerregistry.fi/statistics, [25]
				*First reminder: reminder letter*					
x		x	x	Reminder letter	0.75	30,000	22,500		
x		x	x	Pap-smear for attendees (27 %)	29.80	8,100	241,380		[12, 14, 25]
				Referral for diagnostic colposcopy		110		The pooled rate of referrals among attendees by reminder letter in the studies; 1.4 % (50/3,688)	[12, 14]
x		x	x	No CIN diagnosis^a^	1,047.10	55	57,591		[25]
x		x	x	CIN1 case, inlc. treatment and follow-up^b^	1,863.40	11	20,497	The pooled yields of CIN lesions among participants after 1st reminder in the studies; 0.1 % for CIN1 (6/4,444) and 0.5 % for CIN2+ (24/4,444)	[12, 14, 25]
x		x	x	CIN2+ case, inlc. treatment and follow-up^b^	2,512.00	44	110,528		[12, 14, 25]
				*First reminder: self-sampling*					
	x			Sampling device^c^	2.00/6.50	30,000	60,000–195,000		
	x			Information/invitation letter	0.75	30,000	22,500		
	x			Outbound mailing and other logistic costs^c^	2.90	30,000	87,000		
	x			Inbound mailing costs for attendees (32 %)	1.20	9,600	11,520		[12]
	x			HrHPV analysis for attendees (32 %)	20.00	9,600	192,000		[12]
	x			Response letter for attendees	0.75	9,600	7,200		
				Invitation for follow-up Pap-smear (hrHPV-positives)		1,152		12 % test positivity rate	[12, 14]
	x			Follow-up Pap-smear	29.80	945	28,161	82 % compliance to follow-up	[12, 14, 25]
				Refererral for diagnostic colposcopy		210		The rate of colposcopies after Pap-smear triage in the first study, 22.2 % (6/27 Pap-smears taken)	[12]
	x			No CIN diagnosis^a^	1,047.10	156	163,348		[25]
	x			CIN1 case, inlc. treatment and follow-up^b^	1,863.40	11	20,497	The yield of CIN lesions among attendees after 1st reminder in the studies (see above), but accounting for 18 % loss in follow-up	[12, 14, 25]
	x			CIN2+ case, inlc. treatment and follow-up^b^	2,670.30	43	114,823		[12, 14, 25]
				*Second Reminder: self-sampling*					
		x	x	Sampling device^c^	2.00/6.50	21,900	43,800-142,350		
		x	x	Information/invitation letter	0.75	21,900	16,425		
		x	x	Outbound mailing and other logistic costs^c^	2.90	21,900	63,510		
		x	x	Inbound mailing costs for attendees (21 %)	1.20	4,599	5,519		[12, 14]
		x	x	HrHPV analysis for attendees (21 %)	20.00	4,599	91,980		[12, 14]
		x	x	Response letter for attendees	0.75	4,599	3,449		
				Follow-up: pap-smear triage					
				Invitation for follow-up Pap-smear (hrHPV-positives)		552		12 % test-positivity rate	
		x		Follow-up Pap-smear	29.80	436	12,993	79 % compliance to follow-up	[12, 14, 25]
		x		Referral for diagnostic colposcopy		83		Pooled rate of referrals after Pap-smear triage in the studies, 19.1 % (9/47 Pap-smears taken)	[12, 14]
		x		No CIN diagnosis^a^	1,047.10	25	26,178		[25]
		x		CIN1 case, inlc. treatment and follow-up^b^	1,863.40	27	50,312	The pooled yield of CIN lesions among test positive participants compliant to follow-up in the studies; 6.1 % for CIN1 (7/114) and 7.0 % for CIN2+ (8/114)	[12, 14, 25]
		x		CIN2+ case, inlc. treatment and follow-up^b^	2,670.30	31	82,779		[12, 14, 25]
				Follow-up: direct colposcopy					
				Referral for direct colposcopy for hrHPV-positives		552		12 % test-positivity rate	[14]
			x	No CIN diagnosis^a^	1,047.10	432	452,347	90 % compliance to follow-up	
			x	CIN1 case, inlc. treatment and follow-up^b^	1,863.40	30	55,902		[12, 14, 25]
			x	CIN2+ case, inlc. treatment and follow-up^b^	2,670.30	35	93,461		[12, 14, 25]

^a^ Includes also minor abnormalities without CIN diagnosis, average cost per case including diagnostic procedures and follow-up

^b^ Average costs per CIN case, including diagnostic confirmation, possible treatment and follow-up

^c^ In the estimate with a possibility to opt-out from self-sampling, units needed is 85 % of all non-attendees

The original studies this analysis is based on were approved by the Ethical committee of the Hospital District of Helsinki and Uusimaa (430/E9/07 HUS and 79/13/03/03/2011) and National Institute for Health and Welfare (THL/1465/6.02.00/2013). In the first randomized study the participants gave their written consent for their samples to be used in the study [12, 13]. In the second study no written consent was required for the use of the screening-related data [14]. The data was anonymized prior to analysis in both of the studies. In the current analysis, no individual-level data was used and no extra permissions for the use of data were required.

## Results

### Self-sampling vs. reminder letter as a first reminder

Considering only the costs of invitations and a primary screening test, the cost per extra screened woman by a reminder letter was 33 euros and by self-sampling 38–68 euros depending on the price of the sampler, HPV-analysis and whether an opt-out strategy was used (Table 2). Assuming similar CIN detection rates among the participants of the respective interventions, the cost of an extra detected and treated CIN2+ lesion was approximately 10,300 euros with a reminder letter, and 15,300–21,800 euros by self-sampling.

**Table 2:** Total resource required for screening and cost per detected and treated CIN2+ lesion (in euros). Estimates in self-sampling as a 2nd reminder for two different follow-up strategies after a hrHPV-positive self-taken sample. In the main estimate, sampler price is at 2.0 euros and cost of hrHPV-analysis at 20.0 euros. For breakdown of costs, see Table 1

	Primary invitation only	One reminder		Two reminders	
		Reminder by self-sampling	Reminder by reminder letter	Reminders by reminder letter & self-sampling	Reminders by reminder letter & self-sampling
		Follow-up^a^: Pap-smear triage, compliance 82 %		Follow-up^a^: Pap-smear triage, compliance 79 %	Follow-up^a^: direct colposcopy, compliance 90 %
Primary invitation					
Cost per screened woman (prim. testing^b^)	31	31	31	31	31
Total costs	3,210,443	3,210,443	3,210,443	3,210,443	3,210,443
Cost per treated CIN2+	15,288	15,288	15,288	15,288	15,288
1st reminder					
Cost per screened woman (prim. testing^b^)		43	33	33	33
Total costs		707,049	452,496	452,496	452,496
Cost per extra treated CIN2+		16,443	10,284	10,284	10,284
2nd reminder					
Cost per screened woman (prim. testing^b^)				53	50
Total costs				396,944	826,393
Cost per extra treated CIN2+				12,805	23,611
*Total costs of screening*					
Cost per screened woman (prim. testing^b^)	31	33	31	33	32
Total costs	3,210,443	3,917,492	3,662,939	4,059,883	4,489,332
Cost per treated CIN2+	15,288	15,484	14,421	14,245	15,534
Total cost increase by reminders	-	22 %	14 %	26 %	40 %
Achieved attendance rate	70.0 %	79.4 %	78.1 %	82.6 %	82.6 %
Increase in CIN2+ detection		20 %	21 %	36 %	38 %
Additional analysis:					
Self-sampling opt-out rate 15 %					
Cost per screened woman (prim. testing^b^)		41		49	47
Cost per detected CIN2+ (by reminder)		15,930		12,285	23,175
Sampler price = 6.5 euros					
Cost per screened woman (prim. testing^b^)		58		75	72
Cost per CIN2+ lesion (by reminder)		19,583		15,984	26,427
Cost per CIN2+ lesion (total)		16,018		14,591	15,875
Cost of HPV-analysis = 15–30 euros					
Cost per screened woman (prim. testing^b^)		38–54		48–63	45–60
Cost per CIN2+ lesion (by reminder)		15,327–18,676		12,063–14,288	22,954–24,925
Cost per CIN2+ lesion (total)		15,294–15,864		14,165–14,407	15,454–15,693
Sampler price = 6.5 euros & cost of HPV-analysis = 30 euros					
Cost per screened woman (prim. testing^b^)		68		85	82
Cost per CIN2+ lesion (by reminder)		21,815		17,467	27,741
Cost per CIN2+ lesion (total)		16,397		14,752	16,034
Compliance to Pap-smear triage = 70 %					
Cost per CIN2+ lesion (by reminder)		18,553		13,966	
Cost per CIN2+ lesion (total)		15,766		14,377	
Compliance to Pap-smear triage = 70 % & cost of HPV-analysis 30 euros & sampler price = 6.5 euros					
Cost per CIN2+ lesion (by reminder)		24,970		19,320	
Cost per CIN2+ lesion (total)		16,705		14,892	
CIN yield by self-sampling +20 % compared to reminder letter (1.2 x yield by reminder letter)					
Cost per CIN2+ lesion (by reminder)		14,150			
Cost per CIN2+ lesion (total)		15,066			
CIN yield by self-sampling +50 % compared to reminder letter (1.5 x yield by reminder letter)					
Cost per CIN2+ lesion (by reminder)		11,644			
Cost per CIN2+ lesion (total)		14,421			
Reminder letter participation rate = 14 %					
Cost per CIN2+ lesion			10,693		
Increase in costs by reminders			7.7 %		
Increase in CIN2+ detection (total)			11.0 %		

Assuming 70 % attendance rate with the primary invitation, 27/32 % attendance with first reminder (reminder letter/self-sampling, respectively), and 21 % attendance rate with self-sampling as second reminder

^a^ Follow-up for women with a HPV-positive result from the self-taken sample

^b^ including only costs of invitations, primary screening test and possible triage testing

### Self-sampling as a second reminder after two invitation letters

The cost per extra screened woman (invitations and primary and triage testing only) by self-sampling as a second reminder was 48–85 euros (Table 2). The price to detect and eradicate a CIN2+ lesion by self-sampling ranged from 12,100 to 27,700 euros. When self-sampling was used as a second reminder with a sampler price of 2 euros and a Pap-smear triage, the cost of a CIN2+ lesion detected by self-sampling (12,800 euros) was not higher than one detected in routine screening after primary invitation only (15,300 euros) or after two invitations (14,400 euros). This applied also to higher HPV-analysis cost of 30 euros (14,300 euros).

If a Pap-smear triage was used for women with a HPV-positive result in the self-taken sample the addition of two reminders to the invitation protocol did not increase the price per treated CIN2+ lesion in the entire screened population (15,300 euros with only primary invitation vs. 14,200-14,900 euros after reminders). With direct colposcopy referral for HPV-positive women the cost per lesion in the entire population did increase slightly (15,500–16,000 euros).

## Discussion

In our model population of 100,000 women with original participation rate at 70 %, total participation increased to 78–79 % by one reminder and to 83 % by two reminders (reminder letter and consequent self-sampling) and CIN2+ lesion yield increased by 21 % and 36-38 %, respectively, when total costs increased by 14–29 % (one reminder) and 26–44 % (two reminders).

As a first reminder, self-sampling was more expensive than a reminder letter. Firstly, the cost per extra participating woman was higher (Table 2). Secondly, assuming similar CIN2+ detection rates by self-sampling and reminder letters, or even a 20 % or 50 % higher detection rate by self-sampling, the cost per treated CIN2+ lesion by self-sampling was higher than with a reminder letter.

When self-sampling was used as a second reminder with lower sampler price and a Pap smear triage, the cost of a CIN2+ lesion detected by self-sampling was not higher than one detected in routine screening. This applied also to a higher cost of the HPV-analysis at 30 euros (Table 2). A lower follow-up compliance rate of 70 % compliance would increase the cost per detected CIN2+ lesion, but with lower sampler price and lower price of HPV-analysis it would still be lower than the price per lesion with primary invitation only (14,000 versus 15,300 euros). Further, with Pap-smear triage, adding self-sampling to the invitation protocol did not increase the overall cost per treated CIN2+ lesion in the study population as a whole—even with higher sampler price, higher price of HPV-analysis or lower (70 %) compliance to follow-up. This indicates more lesions could be detected and treated with same cost per lesion.

Strategies to improve participation have been considered cost-effective in improving the current cervical cancer screening programs [27]. Further, as shown by a recent modeling study, strategies that include offering self-sampling to non-attendees generally are cost-effective, unless hrHPV-testing on self-samples would be substantially less accurate and regularly attending women should switch to self-sampling, or the response of non-attenders to use self-samplers would be poor [28]. A recent Swedish randomized trial registered costs of a self-sampling approach and by applying a ratio of six treated CIN2+ lesions to avert one cancer, the authors concluded this intervention would likely be cost-saving and at least cost neutral [22]. In a Dutch study the cost per extra CIN2+ lesion detected by self-sampling among original non-attendees were in the same range as those calculated for conventional cytological screening in the Netherlands [29]. In France, Haguenoer et al. calculated incremental cost-effectiveness ratios (ICERs) per extra screened non-attending woman by self-sampling (63.2 euros) and by a reminder letter (77.8 euros) compared to those screened without extra interventions in France. The authors concluded that the self-sampling strategy could be cost-effective as compared with a reminder letter, as additional costs of the self-sampling strategy were offset by the substantial difference in participation [26]. Assuming there would have been no screening attendance in the reminded population without the interventions, the costs per extra screened woman calculated in our study (33 euros for reminder letter and 43 euros for self-sampling, 38/54 if HPV-analysis price is set at 15/30 euros) are comparable to the ICERs calculated for the French setting. The difference in attendance with the interventions was so noticeable in the French study (23 vs. 10 %) that it compensated for the additional costs of the self-sampling strategy. Based on our cost evaluation, this was not the case in the Finnish setting.

In Finland, extensive opportunistic screening occurs beside and independent of the organized program. The overall 5-year coverage of any screening test has been estimated at approximately 90 % among women in screening ages [24]. Indeed, although offering self-sampling to non-attendees has had a good impact on screening attendance, its effect on overall test coverage has been modest, as only 21–29 % of self-sampling participants reported no Pap-smears within the last 5 years and could thus be regarded under screened [12–14]. If the cost per woman screened by self-sampling is calculated only for the previously under screened self-sampling participants, it rises up to 183–259 euros (sampler price 2–6.5 euros).

On the other hand, total annual costs of all screening tests in Finland have been estimated at 22.4 million euros, of which 78 % comprised of opportunistic screening services with significant over screening among some women and lower cost-effectiveness than that of the organized program [24, 25]. The price of an opportunistic Pap-smear is clearly higher than one taken within the organized program, 54–82 vs. 30 euros [24]. The price of an opportunistic screening test is also higher than the combined price of the invitational system and primary testing per participant in the current estimate, even with one or two reminders (31–34 euros). Thus minimizing the number of opportunistic smears and increasing attendance in organized screening by reminders and a self-sampling option could decrease the overall costs of screening. Importantly, the health benefits of screening would hopefully be more evenly and equally distributed. Self-sampling offers a different approach to screening, in practical and emotional aspects, and can thus help to tempt the current non-attendees of organized screening to take part in the program [30].

We previously showed that reminder letters with assigned appointments result in a two-fold attendance rate (14 vs. 28 %) compared to open invitations that require more of an initiative from the women [14]. However, if reminder letters are used by the Finnish municipalities, they are often open ones (no appointment). When the participation rate of reminder letters was set at a lower level of 14 %, the increase in cost reduced to 8 %, but the total participation rate would only rise up to 74 % and increase in CIN2+ yield would naturally be lower. However, the price per participant was still lower than by self-sampling as a first reminder (Table 2). The previous results and current estimate thus imply that as a first reminder, reminder letters with pre-assigned appointment times and places are the most effective choice.

The follow-up strategy used for hrHPV-positive self-sampling participants has a noticeable impact on the immediate total costs of self-sampling, but it also might have an impact on the rate of detected CIN2+ lesions, as follow-up by direct colposcopy would most likely result in higher compliance to follow-up. In these estimates however, the assumed higher rate of CIN2+ detection in the direct colposcopy approach did not even out the higher costs in terms of cost per eradicated lesion. New triage methods by molecular marker analysis of the self-taken samples show promising results in directing the right women to colposcopic examinations without the extra screening visit a Pap-smear triage requires [31].

At lower sampler prices, the resulting savings of an opt-out approach are limited. However, opt-out reduces waste and is thus recommendable at all sampler prices. An opt-in strategy where samplers are only mailed to those who express an interest would most likely reduce the costs of self-sampling. It does, however require more of an initiative from the women who are already harder to reach, and has generally resulted in lower attendance rates, especially if contact letters offering self-sampling are limited to only one or two [2, 21]).

### Strengths and limitations

Both studies used for this cost analysis were embedded in routine screening. The first study was conducted in a setting where original attendance with primary invitation was lower (63 %) and the second one in a setting with higher (73 %) level of attendance than the national average of 70 %. They both achieved similar rates of participation by interventions in large and diverse populations and resulting participation rates can thus be regarded reliable. Also a similar trend of higher CIN yield among original non-attendees was seen in both studies. This gives support to the generalizability of these findings.

In the original studies, the follow-up protocol was to refer women with a hrHPV-positive self-taken sample aged 40 years or older directly to a colposcopy and invite women aged less than 40 years for a triage Pap-smear. Due to the more complicated nature of the approach, and the unlikelihood of it being used in routine practice, this approach was not used here. Thus in the Pap-smear triage the number of further referrals for colposcopies (HPV-positive and abnormal cytology) was estimated based on the colposcopy rate of women under 40 years who were invited for triage Pap-smear in the studies, although the referral rate might be age dependent in reality.

Further, the cost used for colposcopic examinations and follow-up for women with no CIN diagnosis was quite high, 1,047 euros (vs. cost of colposcopy and biopsies alone, 300–500 euros). Women referred for colposcopy on the basis on HPV-positivity without cytological changes (direct colposcopy after self-sampling) might need less intensive follow-up than women referred due to cytological abnormalities, and for them the used average cost might be an over estimation, but for comparability the same cost was used for all colposcopy cases without CIN diagnosis.

In the absence of information on longer term health benefits achieved by the increase in attendance, such as cancer reduction or life years gained, this evaluation focused on cost per treated CIN2+ lesion, which is clearly an incomplete surrogate. As a further limitation, these studies were planned to observe differences in attendance rates, not in CIN yields. Thus, the resulting CIN yields remained small, and are susceptible for chance, depending for example upon diagnostic workup between laboratories. The estimated price per detected and treated CIN2+ lesion should thus be interpreted with caution.

A crucial aspect of HPV-testing on self-taken samples is the heterogeneity between testing methods. A recent meta-analysis concluded that when PCR-based HPV-tests were used on self-taken samples the relative sensitivity and specificity were similar to clinician-collected samples. However, when less sensitive signal based assays such as HC2 were used, sensitivity of self-sampling was lower, and often also specificity was lower. Both studies used in this estimation used HC2 as the analysis method, and referral rates, CIN yields and resulting costs might vary for more sensitive methods [32]. The clinical implications of the used follow-up strategy for self-sampling HPV-positive women should be further investigated for more precise cost-efficacy analyses on this aspect. Further, outcome evaluations, also with societal perspectives (e.g. taking into account the patient’s time costs in acquiring health care services), are needed for final health economic analyses.

## Conclusions

As a first reminder to non-attendees after primary invitation, self-sampling might result in slightly higher attendance rate, but the reminder letter with a pre-booked screening appointment is still most likely a better choice in terms of cost per extra screened woman and possibly also cost per treated CIN2+ lesion.

When self-sampling was used as a second reminder with lower sampler price and a triage Pap-smear instead of direct colposcopy for HPV-positive women, the eradication of an extra CIN2+ lesion by self-sampling was not more expensive than in routine screening. Based on this surrogate information it thus seems that the higher prevalence of precursors in the non-attending population might even out the higher costs of self-sampling in terms of price per treated precursor lesion. The implementation of self-sampling as a second reminder to non-attendees is worth exploring further.
